# Patient experience in National digital health platforms: Evidence from patient-generated feedback in Saudi Arabia

**DOI:** 10.1371/journal.pone.0360416

**Published:** 2026-10-22

**Authors:** Abdulmohsen Saud Albesher

**Affiliations:** Department of Information Systems, College of Computer Sciences and Information Technology, King Faisal University, Al-Ahsa, Saudi Arabia; Islamic World Science & Technology Monitoring and Citation Institute (ISC), IRAN, ISLAMIC REPUBLIC OF

## Abstract

National digital health platforms are becoming fundamental components of modern healthcare delivery; however, patient experience is commonly assessed using overall ratings or sentiment analysis, providing limited understanding of the specific dimensions that influence digital healthcare quality and service improvement. This study aimed to develop a multidimensional framework for understanding patient experience in a national digital health platform, examine variation across healthcare services, investigate changes over time, and identify evidence-based improvement priorities. A qualitative analysis was conducted using 97,034 Google Play reviews of Sehhaty, the Saudi national digital health platform, posted between 2019 and 2026. Reviews were analyzed using an inductive coding approach to develop a patient experience framework, followed by comparative analyses across healthcare services and longitudinal analyses across successive stages of platform development. Nine patient experience dimensions were identified. Patient experience differed considerably across healthcare services, indicating that improvement priorities were service-specific rather than uniform across the platform. Longitudinal analyses showed a shift from predominantly technical concerns during early deployment toward challenges involving service accessibility, integration, continuity of care, as the platform matured. These findings enabled the identification of service-specific priorities for improving digital healthcare delivery. Patient experience in national digital health platforms is multidimensional, service-dependent, and evolves alongside platform maturity. The proposed framework extends approaches based primarily on ratings or sentiment by transforming large-scale patient-generated feedback into actionable evidence for healthcare quality improvement and continuous evaluation of patient-centered digital health services.

## 1. Introduction

Digital health platforms have become an important component of contemporary healthcare systems [1,2]. They enable patients to access a wide range of services through mobile applications, including appointment scheduling, vaccination services, electronic health records, prescription management, and teleconsultation. Their expansion has reshaped interactions between healthcare organizations and beneficiaries and has become increasingly central to national digital transformation initiatives [3–5]. As a result, ensuring a positive patient experience has become a major objective for healthcare providers, application developers, and policymakers.

In Saudi Arabia, digital health has expanded substantially under Vision 2030 [6]. Sehhaty has emerged as one of the country’s main national digital health platforms [7,8]. Sehhaty integrates multiple healthcare services into a single mobile platform and supports activities such as appointment management, access to medical reports, review of personal health records, and vaccination-related services [8,9]. Comparable large-scale patient-facing digital health platforms also exist internationally, but they vary in structure, functions, and scope of integration. England’s NHS App provides a national ‘digital front door’ for services such as appointment booking, prescription ordering, access to records, and vaccination certification, yet its functions are partly mediated through local general practice systems and are therefore not always available uniformly across users [10]. Brunei’s BruHealth similarly operates at national scale and links the patient-facing app to centralized health records and vaccination services, whereas Türkiye’s e-Nabız is organized primarily as a national personal health record system with broad interoperability across health facilities and public institutions [11,12]. These examples show that integrated national platforms are an established international model, but their architectures and service configurations differ, making Sehhaty a relevant case for examining patient experience within a single government-led mobile platform that combines multiple healthcare functions. As the platform has evolved across successive releases, users have continuously documented their experiences through public app-store reviews, creating a large source of real-world evidence on expectations, frustrations, and perceived service quality.

User-generated reviews are increasingly valuable for evaluating healthcare applications because they capture authentic experiences outside controlled study settings [13]. Unlike questionnaires or usability studies with relatively small samples, app-store reviews provide large-scale feedback from diverse users over extended periods [14,15]. Prior work shows that such reviews can reveal usability problems, software bugs, privacy concerns, account-access failures, feature requests, and other determinants of user satisfaction in mHealth applications [15,16]. Reviews also provide richer context than rating scores alone, which are often insufficient for understanding the specific causes of satisfaction or dissatisfaction [15,17]. Prior app-review research has established the value of large-scale user reviews for identifying recurrent concerns such as usability problems, technical failures, privacy issues, content quality, support needs, and feature requests across diverse mHealth domains [14–19]. However, this literature has been dominated by sentiment classification, rating analysis, topic modeling, or broad quality assessment, with less attention to using reviews to develop a multidimensional account of patient experience in relation to healthcare services and care processes [14,15,18,19]. This limitation matters because digital health platforms are not single-purpose tools; they combine heterogeneous healthcare functions, and the experience requirements of those functions can differ by task, interaction pattern, and care context [14,20]. Existing digital patient experience work also shows that experience is shaped by multiple domains beyond interface usability alone, including functionality, organizational context, and patient capability.

A second limitation is that patient experience is often analyzed as a single homogeneous phenomenon even though users evaluate different digital health functions differently. Existing review-based studies have mostly examined either broad app subcategories or single-purpose applications, while studies of more unified platforms emphasize cross-cutting problems such as feature overlap, poor customizability, and fragmented user journeys [15,21]. What remains less clear is how patient experience varies across distinct healthcare service categories within one integrated national platform, where records access, appointments, vaccination services, and consultations may impose different expectations and failure points [22,23].

A third limitation is the lack of longitudinal evidence on how patient experience changes as digital health platforms mature. Recent mHealth review studies explicitly identify this limitation and call for analyses that account for time, platform change, and evolving user concerns [24]. Broader digital health research also suggests that maturity is associated with differences in patient experience and service impact [25]. Yet temporal analyses remain uncommon in national public healthcare apps, leaving open the question of whether user concerns shift from basic technical failures, such as login, registration, and stability problems, toward broader service-quality and coordination issues as platforms expand [26]. A fourth limitation is that review mining often stops at descriptive insight rather than producing decision-ready priorities for healthcare organizations and policymakers. Although review studies often provide recommendations for developers or identify unmet needs, they less often translate large-scale review evidence into service-specific priorities for organizational improvement and decision-making [27].

To address these gaps, this study presents a large-scale qualitative content analysis of 97,034 Google Play reviews of the Saudi national digital health platform Sehhaty. Rather than treating app reviews as indicators of overall satisfaction, the study uses patient-generated feedback as a source of evidence for understanding patient experience, evaluating integrated digital health services, and supporting continuous improvement of national health information systems. The study develops an inductively derived Patient Experience Framework consisting of 9 dimensions that characterize beneficiaries’ experiences with digital health services. Building on this framework, the study compares patient experience across multiple healthcare service categories, examines how those experiences evolve across application maturity phases, and identifies service-specific improvement priorities derived directly from beneficiary feedback. Accordingly, this study addresses the following research questions:

- **RQ1:** What dimensions characterize patient experience in digital health applications?
- **RQ2:** How do patient experience dimensions differ across digital health service categories?
- **RQ3:** How do patient experience dimensions evolve across application maturity phases?
- **RQ4:** What improvement priorities emerge from beneficiaries’ reviews across different digital health services?

This study contributes to the health information systems literature in four ways. First, it develops an inductively derived multidimensional framework for understanding patient experience in national digital health platforms using large-scale patient-generated feedback. Second, it demonstrates that patient experience should be interpreted at the level of individual digital health services rather than treating integrated national platforms as homogeneous systems. Third, it provides longitudinal evidence showing how patient experience evolves across successive stages of platform maturity. Finally, it translates routinely generated patient feedback into service-specific priorities that can support continuous evaluation, healthcare quality improvement, and evidence-informed decision making.

The remainder of this paper is organized as follows. The Literature Review examines related work on digital health applications, patient experience, and app review analysis. The Methodology describes the research design and four-stage analytical workflow, followed by the Results addressing the four research questions. The Discussion considers the findings in relation to previous research and digital health practice. Finally, the Limitations and Future Research outline the study constraints and directions for further investigation, followed by the Conclusion.

## 2. Literature review

Previous research reveals four important gaps in understanding patient experience in national digital health platforms. Most review-mining studies still emphasize generic analytics over patient experience, rarely compare services within one national platform, seldom examine change across platform maturity, and infrequently translate reviews into decision-ready priorities. The overall literature supports that framing while also showing that digital patient experience, app-review analytics, and national platform evaluation have each matured along partly separate lines [14,22,28].

### 2.1. Digital patient experience in national digital health platforms

Digital patient experience is now understood as a multidimensional construct shaped by technology, functionality, interaction design, patient capability, and organizational context rather than by usability alone [22]. That broader framing has emerged because digital health now spans apps, portals, telemedicine, records, and service ecosystems, making patient experience inseparable from how care is organized and delivered across digital touchpoints [22]. At the same time, the evaluation literature remains fragmented. Collectively, existing app usability and quality scales are aimed mainly at professionals, with very limited support for patient-facing assessment and little theoretical grounding [29]. More recent questionnaire work has moved toward multidimensional user experience assessment, but standardization remains weak across digital health domains, including virtual reality applications, where diverse instruments still lack conceptual consistency [30]. Even newer patient-oriented tools such as pSUAPP are designed for bounded clinical follow-up contexts rather than for large public platforms that bundle multiple health services into one interface [31].

These issues become more pronounced in national digital health platforms, where patients interact with appointment booking, telemedicine, medication management, records access, and health information inside a single service environment [21,32]. Unified platforms promise accessibility, convenience, and continuity, but they also expose users to failures in interoperability, uneven feature integration, and organizational fragmentation across care functions [9,22]. Saudi studies similarly reflect these challenges. Reviews of national digital health adoption emphasize growing awareness and uptake, yet practitioner studies consistently identify technical barriers, workflow disruption, poor compatibility with existing systems, and training or support limitations as major constraints on effective use [9,33,34]. Earlier studies evaluating Saudi digital health applications primarily focused on usability and user perceptions of individual COVID-19 applications such as Sehhaty, Mawid, Tawakkalna, and Tabaud, highlighting technical and usability challenges but not examining patient experience across multiple healthcare services within one integrated platform [23].

Patient-centered digital healthcare research therefore increasingly treats experience as an implementation and governance issue, not only a design issue. Recent readiness work argues that patient perspectives should be integrated into usability testing, co-creation, clinical protocol design, implementation monitoring, and the use of patient-reported experience measures across the technology life cycle [35]. That shift aligns with broader patient experience scholarship, which argues that digital feedback becomes useful evidence only when captured through systematic frameworks that can support service improvement and health technology assessment [32].

### 2.2. App review analysis in digital health

App-store reviews have become a valuable source of real-world patient feedback because they capture unsolicited experiences from large and diverse user populations over time. Across mental health, maternal health, multiple sclerosis, depression self-management, and general mHealth platforms, review-based studies consistently show that users comment on functionality, technical performance, content quality, privacy, support, personalization, and feature requests [36–38]. Reviews also provide explanatory detail that star ratings alone cannot capture, including why users abandon apps, what kinds of customization they want, and how specific failures interfere with care-related tasks [37,38]. Recent Saudi studies have similarly demonstrated that combining user-review analysis with usability evaluation provides valuable insights into user satisfaction, functionality problems, and design improvements in specialized healthcare applications [39].

Methodologically, this literature combines qualitative interpretation with computational review mining. Similar review-mining approaches have also been successfully applied outside healthcare, demonstrating their usefulness for identifying usability issues across large collections of user reviews [40]. Some studies use inductive thematic or content analysis to identify recurring strengths, weaknesses, and unmet needs in user narratives [36,37,41]. Others apply machine learning, sentiment analysis, topic modeling, or BERTopic to scale classification and uncover dominant themes across tens of thousands of reviews [24,28]. These approaches have made it possible to compare large app ecosystems, identify predictors of satisfaction, and detect feature-level concerns such as usability, content variety, billing, trust, responsiveness, and consultation availability [24,28].

Yet the dominant analytic lens remains narrower than patient experience. Many studies still treat reviews primarily as inputs for sentiment classification, rating prediction, or broad quality categorization rather than as evidence about digitally mediated care experience. Even when thematic analysis is added, the output often remains a list of positive and negative app features instead of a structured account of experience across access, continuity, communication, and information use [28,41,42]. A second limitation is the unit of analysis. Most review-mining studies analyze the application as a single entity, even when users are interacting with different functions that may generate distinct expectations and problems [36,37]. This app-level framing works reasonably well for single-purpose tools, but it is less informative for integrated public platforms that combine appointments, consultation, prescriptions, records, and follow-up services within one digital interface [21].

A third limitation is the relative absence of longitudinal analysis. Several studies explicitly note that user experience predictors may evolve over time and call for temporal designs, but most review-based analyses remain cross-sectional or confined to short review windows [24]. This matters because digital health platforms undergo repeated releases, service expansion, and changing policy contexts, all of which can shift the salience of technical, access, and coordination concerns [14,34]. A fourth limitation is the weak translation from findings to organizational action. Although review studies often provide design recommendations or identify unmet needs, they less often convert large-scale feedback into service-specific priorities that can support organizational improvement and decision-making [24,28]. This gap is increasingly important as patient feedback is being reframed as a source of evidence for quality improvement and learning health systems rather than as a post hoc measure of consumer satisfaction [32].

Research on digital patient experience, mHealth applications, and app-review analysis has expanded considerably during the past decade. Despite this progress, four important gaps remain in understanding patient experience in national digital health platforms. First, existing review-analysis studies are effective at surfacing bugs, usability issues, privacy concerns, and feature requests, but they rarely derive a comprehensive patient experience framework from large-scale beneficiary narratives [28,37]. The contemporary digital patient experience reviews do propose multidimensional constructs, but these are typically synthesized from prior studies rather than induced from large-scale app-review corpora [14]. Second, most studies still evaluate healthcare apps as single systems instead of comparing patient experience across multiple healthcare service categories delivered within one integrated national platform. This leaves limited evidence on how experience differs between access-oriented, record-oriented, consultation-based, and coordination-heavy services inside the same public digital infrastructure [21].

Third, although several studies recommend longitudinal follow-up, relatively few examine how patient experience changes across platform maturity phases and successive releases [24]. Existing maturity and readiness research suggests that patient experience should vary as digital systems evolve, but review-mining studies seldom connect user feedback to platform development trajectories [35]. Fourth, most review-mining studies stop at descriptive themes and do not convert beneficiary feedback into service-specific improvement priorities that can guide healthcare managers, developers, and policymakers [38]. The current study addresses these four unresolved issues by developing a multidimensional patient experience framework from large-scale patient-generated feedback, examining service-level differences within a national digital health platform, investigating changes across platform maturity, and translating patient experiences into evidence that supports continuous evaluation and improvement of health information systems.

## 3. Methodology

This study employed a large-scale qualitative content analysis of user-generated reviews to investigate patient experiences with the Sehhaty mobile application. The study adopted an inductive approach in which patient experience dimensions were identified from review content and subsequently examined across digital health services, application release phases, and user-suggested improvement priorities. The analytical workflow was designed to answer four research questions concerning (RQ1) the major patient experience dimensions, (RQ2) their variation across digital health services, (RQ3) their evolution throughout the application’s development, and (RQ4) the improvement priorities expressed by beneficiaries.

User reviews were collected from the Google Play Store using the Google Play Scraper library. The dataset included all publicly available reviews posted between 2019 and 2026, covering multiple application releases throughout the study period. Each extracted record included the review text, star rating, review date, application version, and other publicly available metadata. After removing empty reviews, the final corpus contained 97,034 textual reviews, representing one of the largest publicly available datasets of user feedback for a Saudi digital health application. This corpus served as the basis for all subsequent analyses.

Before analysis, the dataset was audited for missing review text, duplicate records, invalid ratings, missing dates, and missing application-version information. The extracted dataset contained 97,035 records, including one review with empty text; analyses of review content were therefore based on 97,034 non-empty reviews. Review dates and application-version information were standardized to support subsequent analyses. For RQ2, explicit service references were identified computationally using rule-based dictionaries incorporating Arabic and relevant English terms and representing nine digital health service categories: Appointment Booking, Vaccination Services, Medical Reports/Sick Leave, Prescriptions and Medications, Personal Health Record, Instant Medical Consultation, Pregnancy Follow-up, Premarital Screening, and General Health Service. Reviews matching at least one service category were retained for the service-level analysis, resulting in 6,907 service-related reviews (7.12% of the 97,034 non-empty reviews). Service classification was non-mutually exclusive because a review could refer to more than one service. Reviews without an identifiable service reference were excluded from RQ2 only and remained available for the other analyses. For RQ3, application versions were grouped into major–minor release groups (e.g., 3.2.x) and subsequently aggregated into broader platform maturity phases to facilitate longitudinal analysis while avoiding fragmentation caused by individual patch releases.

Major–minor groups represented by fewer than 200 reviews were excluded from the longitudinal analysis. The retained groups were ordered by median review date and assigned deterministically to six analyst-defined version-progression phases: Early Platform (major version 1), Rapid Scale-up (version 2.0–2.5), Service Expansion (remaining major-version-2 groups), Platform Transition (version 3.0–3.10), Maturation (version 3.11–3.20), and Recent Releases (later major-version-3 groups). Median review dates were used only to order the retained version groups; no calendar-date cutoffs or independently documented development milestones were used to define the phase boundaries.

A hybrid AI-assisted and rule-based computational coding approach was used to analyze the review corpus. During framework development, AI-assisted analysis was used to identify recurring concepts, complaints, requests, and experience-related observations in the review corpus. The resulting preliminary coding structure was reviewed and iteratively refined by the researcher. In particular, generic satisfaction statements, healthcare service categories, specific application features, and patient-experience dimensions were conceptually distinguished, and the framework was revised to minimize overlap between dimensions. This iterative process resulted in nine patient-experience dimensions: System Reliability and Stability, Access to Digital Health Services, Usability and Navigation, Efficiency of Digital Service Delivery, Communication and User Support, Authentication and Account Access, Service Integration and Continuity of Care, Information Accuracy and Transparency, and User Control and Autonomy.

Following framework development, the finalized dimensions were operationalized as rule-based text patterns and applied computationally to the full review corpus using Python. The corpus was predominantly Arabic (92,689/97,034, 95.52%), while 1,702 reviews (1.75%) were in English and 214 (0.22%) contained both Arabic and English text; the remaining 2,429 reviews (2.50%) contained no identifiable Arabic or English alphabetic text. Reviews were analyzed in their original language without translation. Before pattern matching, Arabic text was normalized by standardizing common orthographic variants, removing diacritics and tatweel, and normalizing whitespace. The coding dictionaries incorporated both Arabic and English terms where applicable to support classification of Arabic, English, and mixed-language reviews.Each review was evaluated independently against each dimension using regular-expression matching. Coding was non-mutually exclusive; therefore, a review could be associated with multiple patient-experience dimensions when its content addressed more than one aspect of the experience. This computational implementation enabled consistent application of the finalized coding framework across all 97,034 non-empty reviews. The finalized codebook, including operational definitions, inclusion and exclusion guidance, and illustrative review excerpts for the nine patient-experience dimensions, is provided in Supporting Information S2 Table.

To assess coding reliability, an independent blinded validation was conducted using a coverage-oriented stratified sample of 300 reviews. The sample comprised 150 reviews identified computationally as service-related and 150 reviews without a detected service reference, enabling assessment of both positive detections and potential false negatives. It was additionally enriched to ensure representation of English and mixed-language reviews, positive cases across the patient-experience dimensions, and less frequent digital health service categories. A second researcher independently coded the reviews using the finalized codebook for the nine patient-experience dimensions and digital health service categories without access to the computational classifications. Agreement between the independent human coding and the locked computational classifications was assessed separately for each binary code using overall agreement, Cohen’s kappa with 95% confidence intervals, Gwet’s AC1, and positive and negative agreement. Gwet’s AC1 was included because several codes had low positive prevalence and substantial class imbalance, conditions that can reduce kappa despite high observed agreement. Disagreements were subsequently examined through blinded researcher adjudication, followed by targeted researcher–second-coder consensus for the dimensions showing the greatest disagreement. The independent coding decisions were not used to optimize the computational rules.

The analytical process consisted of four consecutive stages corresponding to the research questions. First, the complete review corpus was analyzed to identify the major patient experience dimensions and their relative prevalence (RQ1). Second, service-related reviews were classified into digital health service categories and cross-tabulated with the identified patient experience dimensions to examine how experience varied across healthcare services (RQ2). Third, application-version information was used to investigate how patient experience dimensions evolved across successive platform maturity phases, enabling longitudinal analysis of changes throughout the application’s development lifecycle (RQ3). Finally, reviews containing explicit recommendations, feature requests, or repeated complaints implying desired improvements were analyzed to identify improvement priorities across digital health services (RQ4).

The findings were initially summarized using descriptive statistics and visualized through heatmaps and longitudinal trend figures. Inferential analyses were subsequently conducted to evaluate service-level and temporal differences. Because the coding scheme was non-mutually exclusive, each patient-experience dimension was analyzed as a separate binary outcome. For RQ2, differences across digital health services were tested using binomial generalized estimating equations with service category as the predictor and review identity as the clustering variable to account for reviews referring to multiple services. Service categories containing fewer than 30 reviews were retained for descriptive reporting but excluded from omnibus inferential testing. For RQ3, differences across the six application maturity phases were tested using logistic regression with maturity phase entered as a categorical predictor, while ordered temporal trends were assessed using logistic regression with maturity phase entered as an ordinal predictor. Odds ratios and 95% confidence intervals were reported for temporal trends. Benjamini–Hochberg correction was applied across the nine patient-experience dimensions to control the false discovery rate. The overall analytical workflow followed four sequential stages, as illustrated in Fig 1.

**Fig 1:**
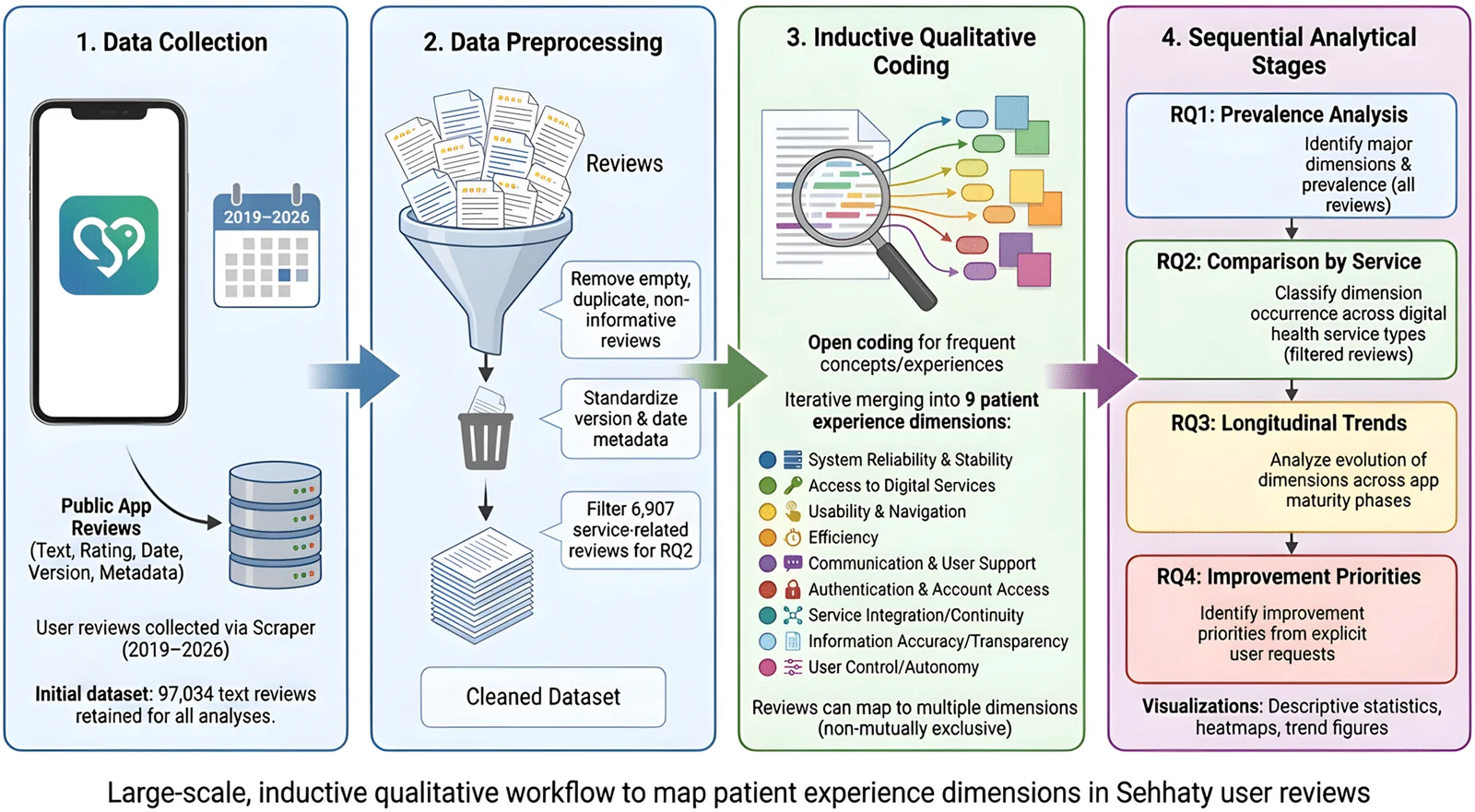
Analytical workflow of the study across the four research questions.

## 4. Results

Table 1 summarizes the characteristics of the review dataset analyzed in this study. A total of 97,034 Google Play user reviews were collected, spanning September 2019 to July 2026, and representing 136 application versions. The dataset includes reviews across all five star-rating levels, with 5-star reviews accounting for the largest proportion (76.7%), followed by 1-star reviews (13.3%), while 2-, 3-, and 4-star ratings collectively represented approximately 10% of the dataset. The large review corpus, broad temporal coverage, and extensive representation of application versions provide a substantial basis for examining beneficiaries’ experiences and identifying recurring themes related to digital health services in Saudi Arabia.

**Table 1:** Characteristics of the user review dataset for saudi digital health services.

Characteristic	Value
Total reviews	97,034
Study period	September 2019 – July 2026
Application versions represented	136
1-star reviews	12,932 (13.3%)
2-star reviews	1,989 (2.1%)
3-star reviews	3,083 (3.2%)
4-star reviews	4,576 (4.7%)
5-star reviews	74,455 (76.7%)

Fig 2 shows the annual distribution of Sehhaty Google Play reviews by star rating from 2019 to 2026. Review activity was limited in the early period, with only 192 reviews in 2019 and 2,541 reviews in 2020, before increasing sharply in 2021, when the dataset recorded 51,839 reviews. This peak suggests that 2021 was the most active year for user feedback in the extracted dataset. Across most years, 5-star reviews formed the largest share of annual feedback. The proportion of 5-star ratings increased from 29.7% in 2019 to 61.5% in 2020, and remained dominant from 2021 onward, accounting for approximately 73.7% to 82.1% of annual reviews between 2021 and 2026. The highest proportion of 5-star reviews was observed in 2025, where they represented 82.1% of all reviews. The figure also shows that 1-star reviews were proportionally highest in 2019, representing 59.9% of reviews that year. However, their relative share declined substantially after 2020, falling to 13.8% in 2021 and remaining comparatively lower in subsequent years. Overall, the figure indicates that while review volume was heavily concentrated in 2021, the rating composition after 2020 was consistently dominated by positive reviews, particularly 5-star ratings.

**Fig 2:**
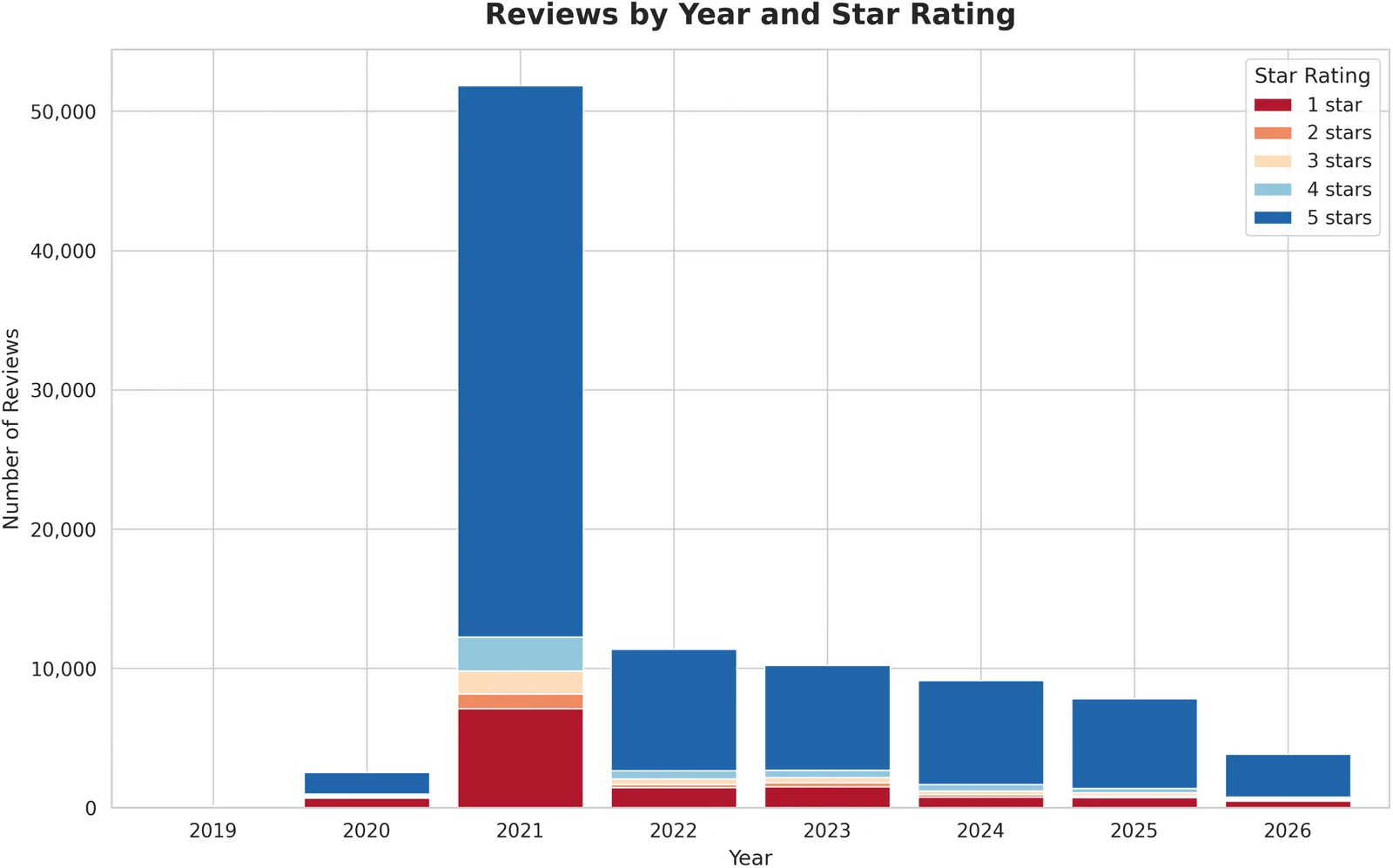
Annual distribution of user reviews by star rating for the national digital health platform (2019–2026).

### 4.1. RQ1. What are the major dimensions of patient experience expressed by beneficiaries when accessing digital health services in Saudi Arabia?

To address RQ1, the review corpus was analyzed to identify the major dimensions of patient experience associated with accessing digital health services in Saudi Arabia. The analysis revealed nine recurring dimensions, as presented in Table 2. Among the identified dimensions, System Reliability and Stability was the most frequently reported, appearing in 7,690 reviews (7.93%). Comments within this dimension primarily described application crashes, loading failures, freezing, and other technical issues that affected beneficiaries’ ability to access services. The second most common dimension was Access to Digital Health Services (6,241 reviews; 6.43%), reflecting challenges related to accessing appointments and other healthcare services through the digital platform. This was followed by Service Integration and Continuity of Care (4,652 reviews; 4.79%) and Communication and User Support (4,510 reviews; 4.65%), indicating that beneficiaries frequently discussed the coordination of healthcare services and the quality of communication and assistance provided during service use. Additional dimensions included Efficiency of Digital Service Delivery (3.24%), Usability and Navigation (3.09%), Authentication and Account Access (2.72%), User Control and Autonomy (1.83%), and Information Accuracy and Transparency (1.36%). Together, these findings demonstrate that beneficiaries’ experiences extend beyond technical functionality to encompass service accessibility, interaction quality, information reliability, and continuity of care.

**Table 2:** Major patient experience dimensions identified from user reviews.

Patient Experience Dimension	Reviews (n)	Percentage (%)
System Reliability and Stability	7,690	7.93
Access to Digital Health Services	6,241	6.43
Service Integration and Continuity of Care	4,652	4.79
Communication and User Support	4,510	4.65
Efficiency of Digital Service Delivery	3,146	3.24
Usability and Navigation	3,001	3.09
Authentication and Account Access	2,636	2.72
User Control and Autonomy	1,774	1.83
Information Accuracy and Transparency	1,324	1.36

#### 4.1.1. Validation of the coding framework.

Independent validation of the coding framework showed overall agreement ranging from 83.3% to 93.3% across the nine patient-experience dimensions (Table 3). Cohen’s kappa ranged from 0.124 to 0.652, with the strongest agreement observed for System Reliability and Stability (κ = 0.652, 95% CI 0.547–0.757) and Access to Digital Health Services (κ = 0.585, 95% CI 0.484–0.687). Gwet’s AC1 ranged from 0.722 to 0.923. Lower kappa and positive-agreement values were observed for Service Integration and Continuity of Care, Information Accuracy and Transparency, and User Control and Autonomy, indicating greater classification uncertainty for these less prevalent and conceptually overlapping dimensions. The overall identification of service-related reviews also demonstrated substantial independent agreement (κ = 0.767, 95% CI 0.694–0.839), while reliability varied across individual service categories; complete service-classification reliability results are provided in Supporting Information S1 Table.

**Table 3:** Independent reliability assessment of patient-experience dimension coding (n = 300).

Dimension	Agreement (%)	Cohen’s κ	95% CI	Gwet’s AC1	Positive agreement (%)	Negative agreement (%)
System Reliability and Stability	88.3	0.652	0.547–0.757	0.825	72.4	92.6
Access to Digital Health Services	83.3	0.585	0.484–0.687	0.722	69.9	88.5
Usability and Navigation	93.3	0.513	0.330–0.697	0.923	54.5	96.4
Efficiency of Digital Service Delivery	85.3	0.391	0.245–0.537	0.807	47.6	91.5
Communication and User Support	90.3	0.531	0.384–0.677	0.879	58.0	94.5
Authentication and Account Access	92.0	0.496	0.324–0.669	0.905	53.8	95.6
Service Integration and Continuity of Care	85.0	0.124	−0.013–0.260	0.820	18.2	91.7
Information Accuracy and Transparency	90.3	0.258	0.079–0.437	0.889	29.3	94.8
User Control and Autonomy	92.0	0.307	0.113–0.501	0.910	33.3	95.7

Cohen’s κ provides chance-corrected agreement. Gwet’s AC1 is additionally reported because several dimensions had low positive prevalence and substantial class imbalance, which can reduce κ despite high observed agreement. Positive and negative agreement are therefore reported separately.

### 4.2. RQ2. Which digital health services are associated with different patient experience dimensions?

To answer RQ2, reviews explicitly referring to digital health services were analyzed to examine how the patient-experience dimensions identified in RQ1 varied across individual services. Fig 3 presents the distribution of patient-experience dimensions within each service category. Inferential analyses confirmed significant differences across service categories for all nine patient-experience dimensions after false-discovery-rate correction (Table 4). The largest cross-service differences were observed for Information Accuracy and Transparency (Wald χ²(6)=1554.60, adjusted p < .001), Access to Digital Health Services (Wald χ²(6)=1645.46, adjusted p < .001), and Service Integration and Continuity of Care (Wald χ²(6)=527.10, adjusted p < .001). Pregnancy Follow-up and Premarital Screening were retained for descriptive reporting but excluded from omnibus testing because of their small numbers of reviews. As shown in Fig 3, Access to Digital Health Services was the dominant experience dimension across several services. It accounted for 97.5% of Appointment Booking reviews and remained the leading dimension for Vaccination Services (63.0%), Pregnancy Follow-up (60.0%), General Health Services (48.4%), Personal Health Records (46.4%), and Instant Medical Consultation (44.4%).

**Table 4:** Inferential comparison of patient-experience dimensions across digital health services.

Patient-experience dimension	Wald χ² (df = 6)	Adjusted p	Prevalence range (pp)
System Reliability and Stability	31.67	<.001	16.34
Access to Digital Health Services	1645.46	<.001	67.58
Usability and Navigation	49.06	<.001	12.67
Efficiency of Digital Service Delivery	74.22	<.001	19.89
Communication and User Support	71.72	<.001	27.02
Authentication and Account Access	49.27	<.001	11.46
Service Integration and Continuity of Care	527.10	<.001	68.98
Information Accuracy and Transparency	1554.60	<.001	75.04
User Control and Autonomy	14.58	.024	6.15

Pregnancy Follow-up (n = 25) and Premarital Screening (n = 7) were excluded from omnibus inferential testing because of their small sample sizes and were retained for descriptive analysis. P values were adjusted using the Benjamini–Hochberg false discovery rate procedure.

**Fig 3:**
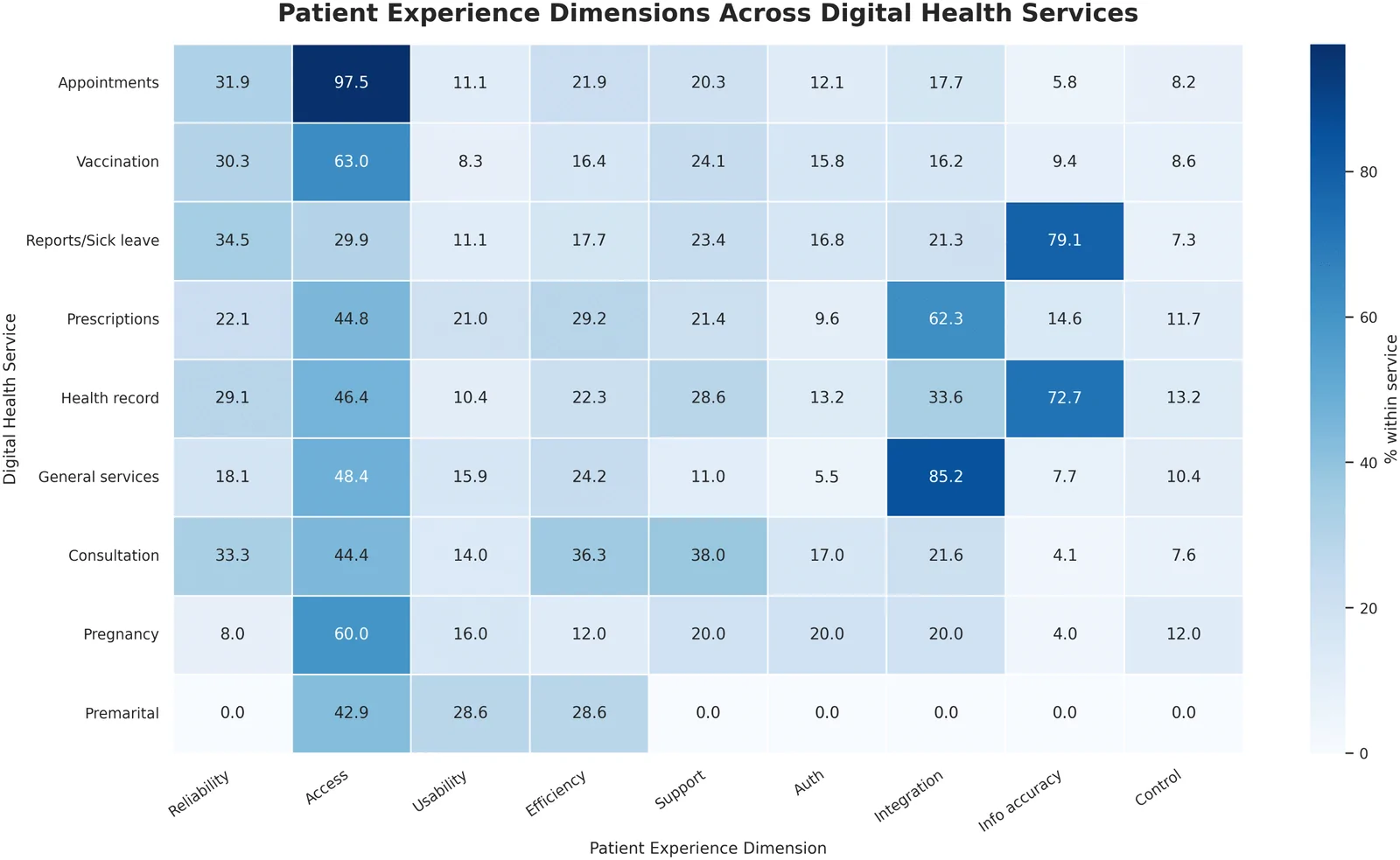
Distribution of patient experience dimensions across digital health services. Percentages represent the proportion of reviews within each service category associated with each patient experience dimension.

Distinct patterns emerged for documentation- and record-oriented services. Medical Reports/Sick Leave and Personal Health Records were predominantly associated with Information Accuracy and Transparency, representing 79.1% and 72.7% of reviews, respectively. Compared with appointment- and vaccination-related services, these services generated substantially more feedback concerning the correctness, completeness, and timeliness of health information presented through the platform. A different experience profile was observed for services requiring coordination across multiple healthcare functions. General Health Services (85.2%) and Prescriptions and Medications (62.3%) were primarily associated with Service Integration and Continuity of Care, indicating that users frequently discussed how effectively information and services were connected across healthcare providers, prescriptions, medical records, and other digital health functions.

Unlike the other services, Instant Medical Consultation exhibited a relatively balanced experience profile. Although Access to Digital Health Services remained the most frequently reported dimension (44.4%), Communication and User Support (38.0%) and Efficiency of Digital Service Delivery (36.3%) were reported at comparable levels. This distribution suggests that consultation services were evaluated through multiple aspects of the user experience rather than being dominated by a single concern. Across all service categories, System Reliability and Stability remained consistently represented, with similar proportions observed across several services, indicating that technical reliability constitutes a cross-cutting issue affecting the broader digital health ecosystem rather than any individual service. In contrast, Information Accuracy and Transparency and Service Integration and Continuity of Care were concentrated within specific services, demonstrating that different digital health services generate distinct patient experience profiles.

### 4.3. RQ3. How did patient experience evolve across platform maturity phases?

To address RQ3, the longitudinal evolution of the five most prominent patient-experience dimensions was examined across successive platform maturity phases. To facilitate visual comparison, each dimension in Fig 4 was normalized to its frequency during the Early Platform phase (100%); inferential analyses, however, were conducted using the underlying review-level binary classifications rather than the normalized figure values. Significant differences across the six maturity phases were observed for all nine patient-experience dimensions after false-discovery-rate correction (Table 5). The most pronounced changes occurred in foundational technical dimensions. System Reliability and Stability declined from 21.60% during the Early Platform phase to 5.59% in the Recent Releases phase, and the ordered trend analysis confirmed a significant decline across maturity phases (OR=0.901, 95% CI 0.882–0.919, adjusted p < .001). Authentication and Account Access similarly decreased from 15.24% to 1.94%, with a significant negative temporal trend (OR=0.779, 95% CI 0.750–0.809, adjusted p < .001). These findings indicate that technical instability and account-access barriers became substantially less prominent across successive platform maturity phases.

**Table 5:** Inferential analysis of patient-experience dimensions across platform maturity phases.

Patient-experience dimension	LR χ² (df = 5)	Omnibus adjusted p	OR per phase	95% CI	Trend adjusted p
System Reliability and Stability	964.46	<.001	0.901	0.882–0.919	<.001
Access to Digital Health Services	181.43	<.001	1.013	0.992–1.035	.218
Usability and Navigation	117.21	<.001	1.071	1.041–1.103	<.001
Efficiency of Digital Service Delivery	85.04	<.001	1.029	1.000–1.060	.056
Communication and User Support	150.95	<.001	0.936	0.912–0.960	<.001
Authentication and Account Access	483.64	<.001	0.779	0.750–0.809	<.001
Service Integration and Continuity of Care	147.82	<.001	1.047	1.023–1.072	<.001
Information Accuracy and Transparency	261.03	<.001	0.852	0.812–0.895	<.001
User Control and Autonomy	41.63	<.001	0.886	0.850–0.924	<.001

Omnibus p values and ordinal-trend p values were separately adjusted using the Benjamini–Hochberg false discovery rate procedure. OR values represent the change in odds associated with a one-phase increase in platform maturity.

**Fig 4:**
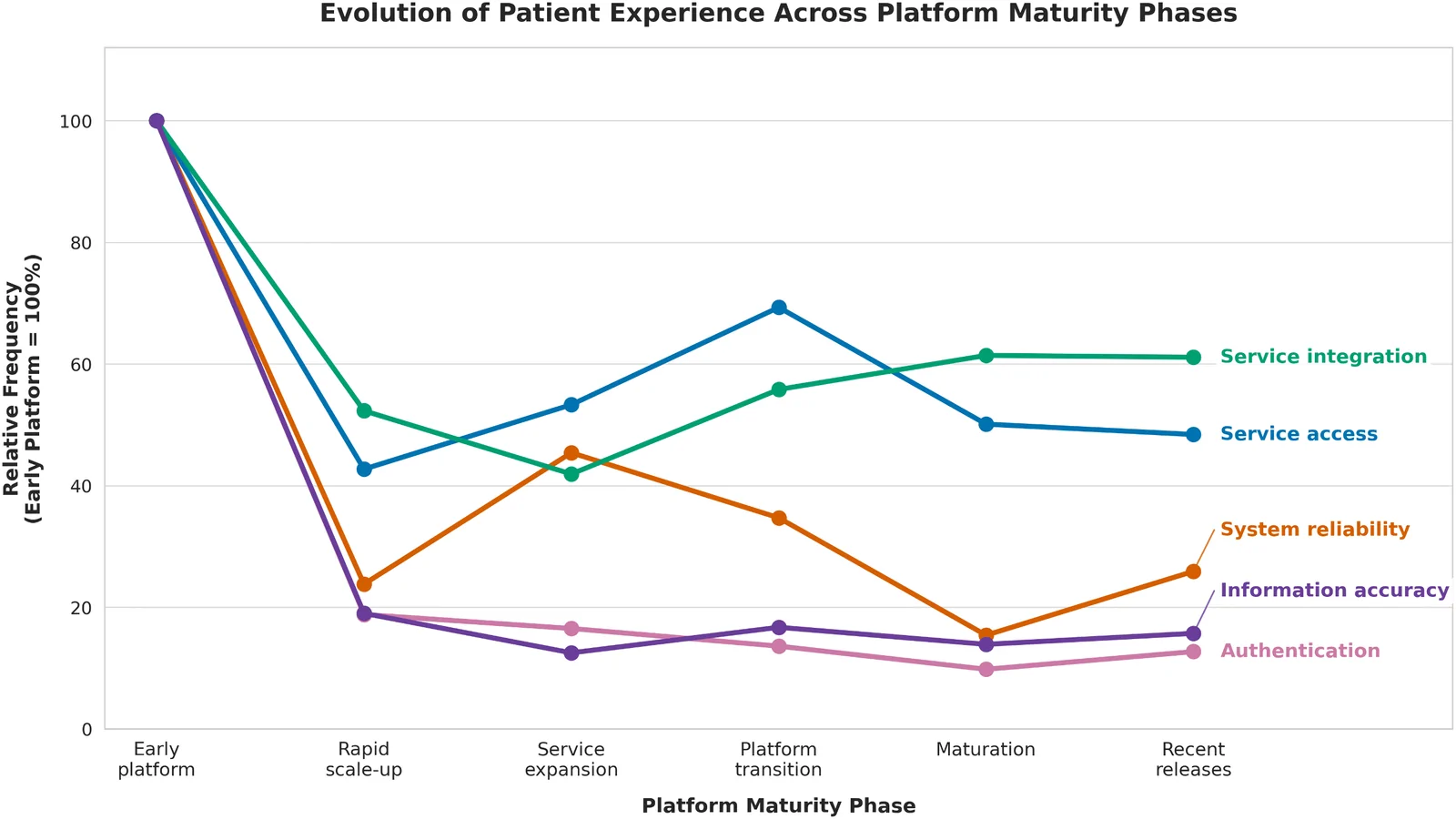
Evolution of the five principal patient experience dimensions across platform maturity phases. Values are normalized to the Early Platform phase (100%) to facilitate comparison of relative changes in patient experience throughout the platform’s evolution.

Fig 4 shows that the largest descriptive shift occurred between the Early Platform and Rapid Scale-up phases, followed by more moderate fluctuations across subsequent phases. Access to Digital Health Services remained a persistent concern throughout platform evolution. Although its prevalence was 12.20% in the Early Platform phase and 5.90% in Recent Releases, the ordered trend was not statistically significant after correction (OR=1.013, 95% CI 0.992–1.035, adjusted p = .218). Access should therefore be interpreted as varying across maturity phases rather than following a consistent monotonic decline. Service Integration and Continuity of Care also remained prominent, reaching 5.88% in Recent Releases and becoming nearly as prevalent as access. The ordered analysis indicated a small positive trend across maturity phases (OR=1.047, 95% CI 1.023–1.072, adjusted p < .001), indicating increasing relative prominence across the ordered phases despite fluctuations between individual phases. In contrast, Information Accuracy and Transparency decreased from 8.21% during the Early Platform phase to 1.29% in Recent Releases, with a significant negative temporal trend (OR=0.852, 95% CI 0.812–0.895, adjusted p < .001). The remaining inferential results, including trends for dimensions not displayed in Fig 4, are reported in Table 5.

### 4.4. RQ4. What improvement priorities do beneficiaries identify for enhancing digital health services?

RQ4 examined the improvement priorities expressed by beneficiaries across different digital health services. The analysis focused on reviews containing actionable requests, recommendations, feature suggestions, or repeated complaints that implied a desired improvement. Fig 5 summarizes the relative priority of improvement themes across service categories. Across the digital health services, the most prominent improvement priorities were System Stability, Appointment Availability, and Support and Guidance. System Stability appeared consistently across several service categories, including Appointment Booking, Vaccination Services, Medical Reports / Sick Leave, Personal Health Record, and Instant Medical Consultation. Appointment Availability was most prominent in services involving scheduling and access to care, while Support and Guidance emerged as a recurring secondary priority across multiple services. The dominant improvement priority varied according to the service category. Appointment Availability was the primary improvement priority for Appointment Booking, Vaccination Services, General Health Services, and Pregnancy Follow-up. System Stability was the dominant priority for Medical Reports / Sick Leave and Instant Medical Consultation. Service Integration was the leading priority for Prescriptions and Medications, whereas Information Accuracy was the dominant priority for Personal Health Record services.

**Fig 5:**
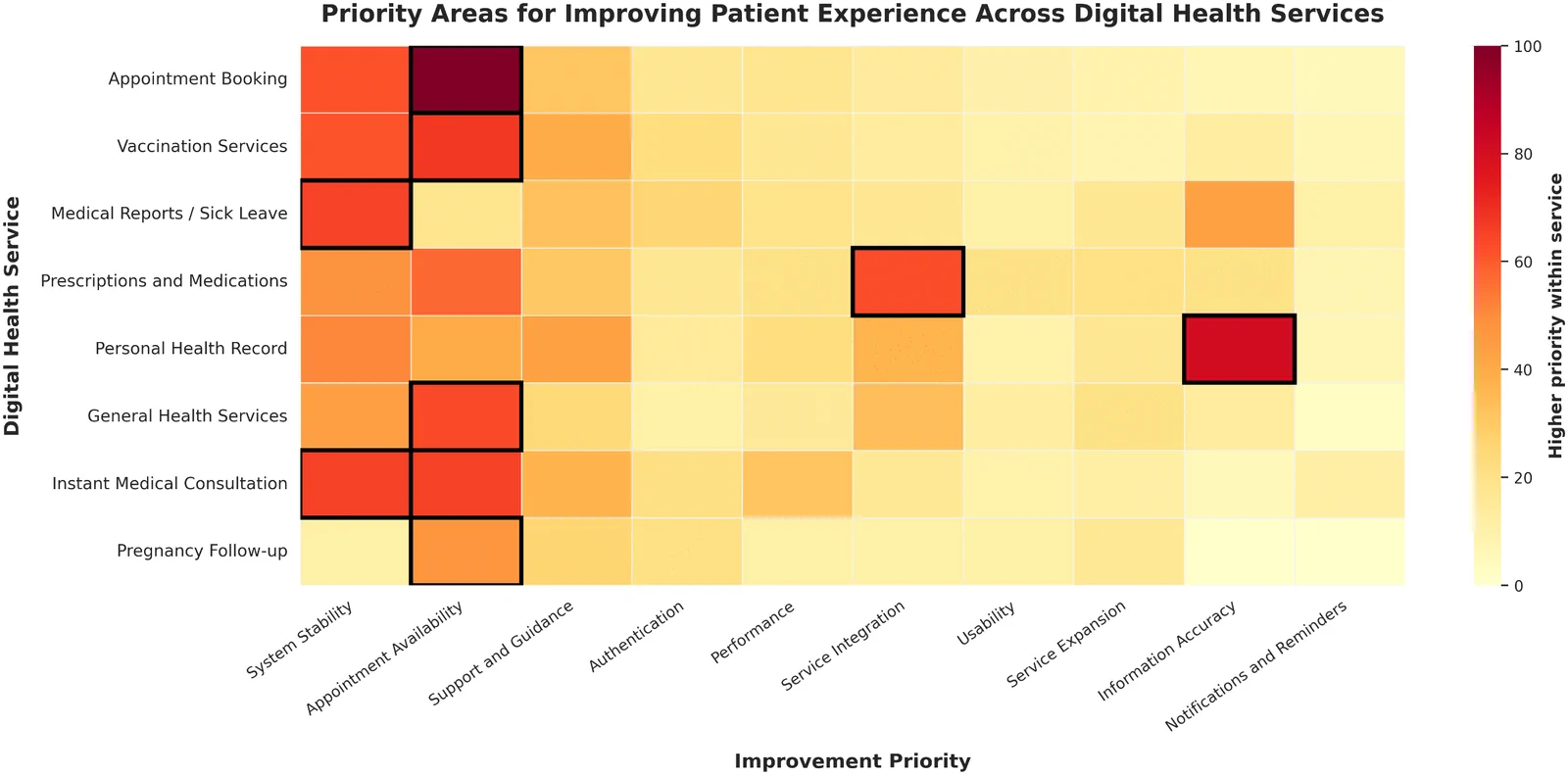
Priority areas for improving patient experience across digital health services. Darker colors indicate higher relative priority within each digital health service. Black outlined cells identify the dominant improvement priority for each service category.

## 5. Discussion

This study demonstrates that patient experience in national digital health platforms represents a multidimensional health information systems construct rather than a simple usability outcome. By analyzing large-scale patient-generated feedback, the findings show that user reviews can provide structured evidence for understanding how patients experience different digital healthcare services and how these experiences can inform continuous service improvement. In this study, user feedback clustered around reliability, access, integration, communication, information quality, and control, supporting the interpretation that patients evaluate digital health platforms as service environments, not merely as software interfaces. This interpretation confirms broader digital patient experience literature, which describes experience as shaped by interacting technological, functional, organizational, and patient-level factors rather than by interface design alone, and extends that literature by showing how these domains become visible in routine patient-generated review data from an integrated national platform [14,29,30].

The service-level patterns further suggest that beneficiaries do not experience a unified national platform as a single homogeneous tool. Instead, different services appear to generate different evaluative logics: access-oriented functions are judged by whether care can be reached, record-oriented functions by the accuracy and transparency of information, and cross-provider services by how well the platform supports coordination and continuity.

The longitudinal findings indicate a shift from early technical and authentication problems toward a later-stage experience profile in which access and integration remained comparatively prominent. Reliability and authentication showed significant declining trends across maturity phases, whereas access varied non-linearly and integration showed a small positive temporal trend. This suggests that platform maturation changes the relative composition of patient-experience concerns, with later-stage experience depending less on basic operability and more on service reachability and continuity across care pathways.

These patterns agree with earlier app-review studies showing that users evaluate digital health apps through technical quality, content, support, customization, and responsiveness, not through sentiment polarity alone [28,37]. They also align with broader digital patient experience research showing that experience extends beyond conventional usability and reflects multiple technological, functional, and patient-centered dimensions [14,29,30]. At the same time, the present study extends prior review-based research because much of the existing literature has examined single-purpose applications or aggregate app experiences rather than comparing multiple healthcare service categories within one integrated national platform [15,21,36–38].

The service-specific patterns identified here therefore suggest that patient experience is partly service-embedded: the relevance of access, information quality, support, or integration depends on the functional and care-related demands of the service being used. This has implications for health information systems evaluation, suggesting that patient experience may be more informative when assessed in relation to specific service functions rather than reduced to platform-wide satisfaction or generic usability measures [14,21,22].

The temporal pattern also extends previous work. Cross-sectional app-review studies have repeatedly identified important experience dimensions, but longitudinal evaluation remains uncommon and further work is needed to examine how user concerns evolve as platforms change [9,24,30]. The observed decline in foundational technical barriers, together with the continued prominence of access and the small positive temporal trend in service integration, is consistent with evidence that digital maturity can change the determinants of user experience and service impact over time [35]. This pattern suggests that as integrated platforms mature, patient-experience concerns can shift from basic operability toward broader issues of care reachability and coordination, which is consistent with digital maturity literature that places increasing emphasis on interoperability, patient-centered care, and system impact across stages of development [22,35]. Collectively, these comparisons indicate that app-store reviews can serve not only as indicators of software quality but also as a valuable source of evidence for understanding patient experience across complex digital health ecosystems [32].

For healthcare organizations, the findings imply that patient experience monitoring should be structured around service lines rather than aggregated at the whole-platform level. Different digital services create different forms of friction, so improvement efforts are likely to be more effective when aligned with the functional demands of appointments, records, consultations, prescriptions, and follow-up rather than with generic app-level metrics [22]. For developers, the practical message is that reliability remains necessary but insufficient. Mature public platforms must support availability, interoperability, accurate information display, and responsive support in ways that reflect the purpose of each service, which is consistent with broader calls for user-centered design, integration, and facilitating conditions that sustain adoption beyond initial rollout [32]. This is particularly relevant in Saudi Arabia, where fragmented feature implementation across apps has been linked to disjointed user experiences and weaker continuity expectations [22]. For healthcare managers, review-derived priorities can function as an operational feedback source for continuous improvement and can inform resource allocation when interpreted alongside implementation evidence. If early-stage complaints center on system stability and login barriers but later-stage complaints center on access bottlenecks and integration gaps, then managerial priorities should evolve accordingly rather than remain fixed on the categories that dominate during initial deployment [9]. For policymakers, patient feedback can support governance, planning, and monitoring of national digital platforms when interpreted alongside adoption and implementation evidence [33].

Beyond extending digital patient experience research, the proposed framework provides a practical analytical approach for health information systems evaluation. Rather than using patient-generated feedback solely to measure satisfaction or identify isolated usability problems, the framework demonstrates how routinely generated user reviews can support service-level and longitudinal evaluation while informing evidence-based governance of national digital health platforms. This perspective also complements previous work describing digital patient experience as a multidimensional construct and ongoing efforts to develop more patient-centered evaluation approaches [14,22,31]. It also provides a practical way to use routine patient-generated feedback to assess how experience varies across services and platform maturity, rather than limiting evaluation to overall satisfaction or usability. Positioning patient experience as a dynamic, service-embedded, and governance-relevant construct helps connect experience theory with implementation and readiness frameworks that increasingly call for patient participation, co-creation, and continuous improvement throughout the technology life cycle [35].

## 6. Limitations and future research

This study has several limitations that should be considered when interpreting the findings. First, the analysis was based exclusively on Google Play reviews and therefore does not capture patient experiences expressed through other app stores, support channels, social media, or direct feedback mechanisms. Second, app-store reviews are voluntarily submitted and may overrepresent users with particularly positive or negative experiences. In addition, the service-level analysis (RQ2) was restricted to reviews containing explicit references to identifiable digital health services; therefore, experiences expressed without naming or clearly identifying a specific service may be underrepresented in the service-specific findings.The available review metadata also did not provide reliable information about users’ demographic characteristics, health conditions, digital literacy, or frequency of platform use. Third, the patient experience framework was developed inductively from reviews of a single national digital health platform. Although the large and longitudinal dataset supported the identification of recurring experience patterns, the framework may reflect features of the Saudi healthcare and digital-service context and therefore requires validation before being generalized to other platforms or countries. In addition, although independent validation showed high overall agreement for most coding dimensions, reliability varied across individual codes, particularly for less prevalent and conceptually overlapping dimensions. Lower positive agreement for Service Integration and Continuity of Care, Information Accuracy and Transparency, and User Control and Autonomy indicates that rule-based classification cannot be assumed to provide error-free identification at the individual-review level. These findings should therefore be interpreted as large-scale patterns in patient-generated feedback rather than exact classifications of every individual review. Finally, user reviews describe perceived experiences but do not provide direct evidence about actual system performance, clinical outcomes, or the organizational causes underlying reported problems.

Future research should validate the proposed framework across other national and institutional digital health platforms and examine whether the identified dimensions remain consistent across healthcare systems and cultural contexts. Combining app-store reviews with interviews, surveys, usability testing, support records, and usage analytics could provide a more comprehensive understanding of the relationship between reported experiences and actual user behavior. Further longitudinal research could also examine how major application updates, service expansions, and policy changes influence patient experience and whether improvement priorities shift as digital health platforms mature.

## 7. Conclusion

This study demonstrates that large-scale patient-generated feedback can provide structured evidence for evaluating patient experience in national digital health platforms. By analyzing more than 97,000 patient reviews collected over seven years, the study developed a multidimensional framework that captures how patients experience digital healthcare services across technical performance, usability, information quality, communication, and healthcare service delivery. The findings further show that patient experience varies substantially across healthcare services and evolves as national digital health platforms mature. Rather than identifying a single set of universal usability issues, the study reveals that different healthcare services exhibit distinct experience profiles and therefore require service-specific improvement strategies. This perspective enables healthcare organizations to move beyond reactive problem solving toward evidence-informed prioritization of digital health improvements based on large-scale patient-generated feedback.

From a scientific perspective, this study contributes a framework for evaluating patient experience in national health information systems, with broader applicability requiring further validation. From a practical perspective, it provides healthcare organizations, developers, and policymakers with a scalable approach for continuously monitoring digital health services, identifying emerging challenges, and prioritizing service improvements throughout platform evolution. As national healthcare systems continue to expand their digital infrastructure, integrating patient-generated feedback into routine evaluation can support more responsive, evidence-informed, and patient-centered digital health ecosystems.

## Supporting information

S1 Table Service Classification Reliability. Independent reliability assessment of the digital health service classifications.
(DOCX)

S2 Table Patient Experience Codebook. Final codebook for the nine patient-experience dimensions, including operational definitions, inclusion and exclusion guidance, and illustrative review excerpts.
(DOCX)

## References

[1] Jeilani A, Hussein A. Impact of digital health technologies adoption on healthcare workers’ performance and workload: perspective with DOI and TOE models. BMC Health Serv Res. 2025;25(1):271. doi: 10.1186/s12913-025-12414-4 39966874 PMC11837663

[2] Zhang L, Bullen C, Chen J. Digital Health Innovations to Catalyze the Transition to Value-Based Health Care. JMIR Med Inform. 2025;13:e57385. doi: 10.2196/57385 39864959 PMC11769777

[3] Stoumpos AI, Kitsios F, Talias M. Digital Transformation in Healthcare: Technology Acceptance and Its Applications. International Journal of Environmental Research and Public Health. 2023;20. doi: 10.3390/ijerph20043407 PMC9963556 36834105

[4] Muthukumarana HTUN, Ranatunga RVSPK. Utilizing Digital Health Technologies To Improve Patient Experience And Care Quality In Global Healthcare: A Systematic Review. IJDHT. 2025;1(2). doi: 10.51137/wrp.ijdht.2025.hmui.45903

[5] Rosenlund M, Kinnunen U, Saranto K. The Use of Digital Health Services Among Patients and Citizens Living at Home: Scoping Review. Journal of Medical Internet Research. 2023;25. doi: 10.2196/44711 PMC10131924 36972122

[6] Aldogiher A, Halim Y. Customer Engagement in Digital Health Transformation as Strategic Change: Evidence from Saudi Arabia’s Vision 2030. Sustainability. 2025. doi: 10.3390/su17188468

[7] Alkhalifah JM, Seddiq W, Alshehri BF, Alhaluli AH, Alessa MM, Alsulais NM. The role of the COVID-19 pandemic in expediting digital health-care transformation: Saudi Arabia’s experience. Inform Med Unlocked. 2022;33:101097. doi: 10.1016/j.imu.2022.101097 36185732 PMC9509531

[8] Alshammari HM, Almutairi RI, Ghazwani AY, Aditya RS. Evaluating user satisfaction and engagement in mHealth: Insights from the Integrated Digital Health Engagement Model (IDHEM). Digit Health. 2025;11:20552076251346698. doi: 10.1177/20552076251346698 40718399 PMC12290358

[9] Alzghaibi H. Examining Healthcare Practitioners’ Perceptions of Virtual Physicians, mHealth Applications, and Barriers to Adoption: Insights for Improving Patient Care and Digital Health Integration. Int J Gen Med. 2025;18:1865–85. doi: 10.2147/IJGM.S515448 40191233 PMC11971970

[10] Reidy C, Papoutsi C, Kc S, Gudgin B, Laverty AA, Greaves F, et al. Qualitative evaluation of the implementation and national roll-out of the NHS App in England. BMC Med. 2025;23(1):20. doi: 10.1186/s12916-024-03842-w 39838384 PMC11752663

[11] Koh KS, Lim HS, Lim J, Wei Y, Minn PW, Wong J. Development and implementation of a national mobile health application: A case study from Brunei. J Glob Health. 2022;12:03083. doi: 10.7189/jogh.12.03083 36538470 PMC9767305

[12] Birinci Ş. A Digital Opportunity for Patients to Manage Their Health: Turkey National Personal Health Record System (The e-Nabız). Balkan Med J. 2023;40(3):215–21. doi: 10.4274/balkanmedj.galenos.2023.2023-2-77 37114621 PMC10175887

[13] Lakhan S. Sentiment and thematic analysis of user reviews for FDA-cleared prescription digital therapeutics: A mixed-methods real-world evidence study. Cureus. 2025;17. doi: 10.7759/cureus.84710 PMC12102580 40416910

[14] Wang T, Giunti G, Melles M, Goossens R. Digital Patient Experience: Umbrella Systematic Review. J Med Internet Res. 2022;24(8):e37952. doi: 10.2196/37952 35925651 PMC9389377

[15] Haggag O, Grundy J, Abdelrazek M, Haggag S. A large scale analysis of mHealth app user reviews. Empir Softw Eng. 2022;27(7):196. doi: 10.1007/s10664-022-10222-6 36246486 PMC9553639

[16] Varghese L, Pai RR, Savitha G, Girisha S, Chetty N. Manual annotation based sentiment analysis of user feedback in health and wellness app reviews. Sci Rep. 2025;15(1):44766. doi: 10.1038/s41598-025-28799-5 41461774 PMC12749540

[17] Haque MDR, Rubya S. An Overview of Chatbot-Based Mobile Mental Health Apps: Insights From App Description and User Reviews. JMIR Mhealth Uhealth. 2023;11:e44838. doi: 10.2196/44838 37213181 PMC10242473

[18] Leong KH, Dahnil DP. Classification of healthcare service reviews with sentiment analysis to refine user satisfaction. International journal of electrical and computer engineering systems. 2022. doi: 10.32985/ijeces.13.4.8

[19] Uncovska M, Freitag B, Meister S, Fehring L. Rating analysis and BERTopic modeling of consumer versus regulated mHealth app reviews in Germany. NPJ Digit Med. 2023;6(1):115. doi: 10.1038/s41746-023-00862-3 37344556 PMC10285024

[20] Orsingher C, McColl‐Kennedy JR, Zaki M, Green T, Varnfield M, Li J, et al. Mobile applications (apps) as service provider actors. Int J Consumer Studies. 2024;48(2). doi: 10.1111/ijcs.13016

[21] Philip BJ, Abdelrazek M, Barnett S, Bonti A, Grundy J. Toward a Unified mHealth Platform: A Survey of Current User Challenges and Expectations. IEEE Access. 2023;11:19876–91. doi: 10.1109/access.2023.3249786

[22] Alharthi S. mHealth applications in Saudi Arabia: current features and future opportunities. Healthcare. 2025;13. doi: 10.3390/healthcare13121392 PMC12193350 40565422

[23] Alghareeb M, Albesher AS, Asif A. Studying Users’ Perceptions of COVID-19 Mobile Applications in Saudi Arabia. Sustainability. 2023;15(2):956. doi: 10.3390/su15020956

[24] Pal S, Biswas B, Gupta R, Kumar A, Gupta S. Exploring the factors that affect user experience in mobile-health applications: A text-mining and machine-learning approach. J Bus Res. 2023;156:113484. doi: 10.1016/j.jbusres.2022.113484 36475057 PMC9715352

[25] Woods L, Dendere R, Eden R, Grantham B, Krivit J, Pearce A, et al. Perceived Impact of Digital Health Maturity on Patient Experience, Population Health, Health Care Costs, and Provider Experience: Mixed Methods Case Study. Journal of Medical Internet Research. 2023;25. doi: 10.2196/45868 PMC10394505 37463008

[26] Mescher T, Hacker RL, Martinez LA, Morris CD, Mishkind MC, Garver-Apgar CE. Mobile Health Apps: Guidance for Evaluation and Implementation by Healthcare Workers. J technol behav sci. 2024;10(2):224–35. doi: 10.1007/s41347-024-00441-7

[27] Jesus TS, Gurung G, Quatman-Yates C. Editorial: Patient Experience Data and Feedback for Quality Improvement and Learning Health Systems. The International Journal of Health Planning and Management. 2025. doi: 10.1002/hpm.3917 39994871

[28] Oyebode O, Alqahtani F, Orji R. Using Machine Learning and Thematic Analysis Methods to Evaluate Mental Health Apps Based on User Reviews. IEEE Access. 2020;8:111141–58. doi: 10.1109/access.2020.3002176

[29] Azad-Khaneghah P, Neubauer N, Cruz MA, Liu L. Mobile health app usability and quality rating scales: a systematic review. Disability and Rehabilitation: Assistive Technology. 2020;16:712–21. doi: 10.1080/17483107.2019.1701103 31910687

[30] Bruni F, Mancuso V, Pedroli E. How to evaluate user experience in digital health? A scoping review of questionnaires in virtual reality applications. Frontiers in Digital Health. 2025;7. doi: 10.3389/fdgth.2025.1561364 PMC12477235 41031121

[31] De Dios López A, Real J, Meza C. Validation of a questionnaire to assess the usability of and user experience with mobile health applications. Healthcare. 2024;12. doi: 10.3390/healthcare12232328 PMC11640890 39684949

[32] Aytekin A, Alan H, Demirel H. Digital health technologies in patient experience literature: A scoping review and future outlook for sustainable digital health interventions. Sustainability. 2025. doi: 10.3390/su17020456

[33] Alfraih AN, Lu H, Singh C. A systemic review on the awareness, adoption, and utilization of digital health in the Kingdom of Saudi Arabia. African and Asian Studies. 2024. doi: 10.1163/15692108-bja10049

[34] Alzghaibi H. Healthcare practitioners’ perceptions of mhealth application barriers: Challenges to adoption and strategies for enhancing digital health integration. Healthcare. 2025;13(5):494. doi: 10.3390/healthcare13050494 PMC11899126 40077056

[35] De La Torre E, Montane C, Rubio O, Sampietro-Colom L, Camacho-Mahamud A, Grau-Corral I. Integrating patient perspectives into the digital health technology readiness framework: Delphi study. Journal of Medical Internet Research. 2025;27. doi: 10.2196/71600 PMC12367350 40835414

[36] Alqahtani F, Orji R. Insights from user reviews to improve mental health apps. Health Informatics J. 2020;26(3):2042–66. doi: 10.1177/1460458219896492 31920160

[37] Biviji R, Williams KS, Vest J, Dixon B, Cullen T, Harle C. Consumer perspectives on maternal and infant health apps: qualitative content analysis. Journal of Medical Internet Research. 2021;23. doi: 10.2196/27403 PMC8444044 34468323

[38] Polhemus A, Simblett S, Dawe Lane E, Elliott B, Jilka S, Negbenose E, et al. Experiences of health tracking in mobile apps for multiple sclerosis: A qualitative content analysis of user reviews. Multiple Sclerosis and Related Disorders. 2023;69:104435. doi: 10.1016/j.msard.2022.104435 36493561

[39] Albesher AS, Alwahib MF. PLoS One. 2025;20(7):e0329029. doi: 10.1371/journal.pone.0329029 PMC12286377 40700412

[40] Alsanousi B, Albesher AS, Do H, Ludi S. Investigating the user experience and evaluating usability issues in AI-enabled learning mobile apps: An analysis of user reviews. IJACSA. 2023;14(6). doi: 10.14569/IJACSA.2023.0140602

[41] Malik T, Ambrose AJ, Sinha C. Evaluating user feedback for an artificial intelligence–enabled, cognitive behavioral therapy–based mental health app (Wysa): qualitative thematic analysis. JMIR Human Factors. 2021. doi: 10.2196/35668 PMC9044157 35249886

[42] Polhemus A, Simblett S, Dawe-Lane E, Gilpin G, Elliott B, Jilka S, et al. Health tracking via mobile apps for depression self-management: qualitative content analysis of user reviews. JMIR Human Factors. 2022;9(4):e40133. doi: 10.2196/40133 PMC9730209 36416875

