# Supplementary material for: Patient experience in National digital health platforms: Evidence from patient-generated feedback in Saudi Arabia
Source: PLoS One. 2026 Oct 22;21(10):e0360416. doi: 10.1371/journal.pone.0360416 (PMC13690221; doi:10.1371/journal.pone.0360416)
Supplement: Supplementary file 1 [file pone.0360416.s001.docx]

**S1 Table. Independent Reliability Assessment of Digital Health Service Classification (n=300).**

| Service classification | Overall agreement (%) | Cohen’s κ | 95% CI | Gwet’s AC1 | Positive agreement (%) | Negative agreement (%) |
| --- | --- | --- | --- | --- | --- | --- |
| Service Related | 88.3 | 0.767 | 0.694–0.839 | 0.767 | 88.5 | 88.1 |
| S1 — Appointment Booking | 94.3 | 0.861 | 0.797–0.925 | 0.904 | 90.1 | 96.0 |
| S2 — Vaccination Services | 90.0 | 0.459 | 0.305–0.614 | 0.878 | 50.0 | 94.4 |
| S3 — Medical Reports / Sick Leave | 99.0 | 0.909 | 0.807–1.000 | 0.989 | 91.4 | 99.5 |
| S4 — Prescriptions and Medications | 98.0 | 0.718 | 0.503–0.932 | 0.978 | 72.7 | 99.0 |
| S5 — Personal Health Record | 96.3 | 0.725 | 0.570–0.879 | 0.958 | 74.4 | 98.0 |
| S6 — Instant Medical Consultation | 99.3 | 0.930 | 0.833–1.000 | 0.993 | 93.3 | 99.6 |
| S7 — Pregnancy Follow-up | 98.0 | 0.394 | 0.012–0.775 | 0.979 | 40.0 | 99.0 |
| S8 — Premarital Screening | 99.0 | 0.662 | 0.304–1.000 | 0.990 | 66.7 | 99.5 |
| S9 — General Health Services | 91.7 | 0.198 | -0.002–0.399 | 0.907 | 24.2 | 95.6 |

**Note.** Cohen’s κ provides chance-corrected agreement. Gwet’s AC1 is additionally reported to account for prevalence-related effects, particularly for service categories with few positive cases. Positive and negative agreement are reported separately because high overall agreement in low-prevalence categories may be driven primarily by agreement on negative classifications.
