# Supplementary material for: Patient experience in National digital health platforms: Evidence from patient-generated feedback in Saudi Arabia
Source: PLoS One. 2026 Oct 22;21(10):e0360416. doi: 10.1371/journal.pone.0360416 (PMC13690221; doi:10.1371/journal.pone.0360416)
Supplement: Supplementary file 2 [file pone.0360416.s002.docx]

**S2 Table. Patient-Experience Coding Framework, Operational Definitions, and Illustrative Review Examples.**

| **Code** | **Patient-experience dimension** | **Operational definition** | **Inclusion guidance / criteria** | **Exclusion or boundary guidance** | **Illustrative review excerpt (original language)** | **English translation** |
| --- | --- | --- | --- | --- | --- | --- |
| **D1** | System Reliability and Stability | Platform functions consistently and dependably without crashes, freezing, loading failures, repeated errors, or interruptions. | System/application failure or instability. | Service is unavailable but system itself works. | 1. التطبيق لايعمل ارجوا حل المشكله | 1. The application does not work. Please solve the problem. |
| **D2** | Access to Digital Health Services | User can locate, reach, obtain, book, or use an intended digital health service. | Cannot reach/find/book/use a service. | Login/account issue only. | 1. لا يوجد مواعيد أسنان في جميع المراكز الصحيه | 1. There are no dental appointments at any of the health centers. |
| **D3** | Usability and Navigation | Ease or difficulty of understanding, navigating, or interacting with the interface. | Confusing interface/navigation or ease of use. | Delay only, without interface/navigation difficulty. | 1. برنامج جدا جدا صعب | 1. The program is very, very difficult. |
| **D4** | Efficiency of Digital Service Delivery | Time, effort, waiting, repeated attempts, or convenience involved in completing a task. | Delays, excessive effort/steps, repeated attempts, time saving. | Crash alone without time/effort consequence. | 1. فكرته نبيله توفر الوقت و الجهد 2. ياخذ وقت طويل لفتح البرنامج | 1. Its concept is valuable; it saves time and effort. 2. It takes a long time to open the application. |
| **D5** | Communication and User Support | Guidance, notifications, explanations, responses, help, or support. | Missing/unclear guidance, support response, notification or communication. | Incorrect records only. | 1. الافضل لو يصل إشعار عند ظهور نتيجة الفحص 2. طلبت استشارة فوريه لصرف علاج الضغط ولكن مع الاسف لم يتم الرد علي | 1. It would be better to receive a notification when the test result becomes available. 2. I requested an instant consultation for blood-pressure medication, but unfortunately no one responded to me. |
| **D6** | Authentication and Account Access | Creating, verifying, entering, recovering, or using an account. | Login, OTP, identity verification, password, registration/account access. | Healthcare-service access when account login works. | 1. لا يرسل رمز التحقق 2. مايفتح حسابي يقول هناك خطأ | 1. The verification code is not sent. 2. My account does not open; it says there is an error. |
| **D7** | Service Integration and Continuity of Care | Connections among services, records, prescriptions, providers, linked family members, or care pathways. | Missing link/synchronization or continuity across functions. | Single isolated interface issue. | 1. يجب ربط جميع المستشفيات على التطبيق بحيث تجد مواعيدك وتحاليلك وادويتك في التطبيق | 1. All hospitals should be linked to the application so that appointments, test results, and medications are available in the app. |
| **D8** | Information Accuracy and Transparency | Correctness, completeness, currency, visibility, or clarity of health/service information. | Incorrect, missing, outdated, inconsistent, or unclear records/results/status. | Navigation difficulty only. | 1. نتائج التحاليل لاتظهر في التطبيق 2. ادويه يجيني غير مصروفه اروح للصيدليه القاها مصروفه | 1. Laboratory test results do not appear in the application. 2. My medications appear as not dispensed, but when I go to the pharmacy I find that they have been dispensed. |
| **D9** | User Control and Autonomy | Ability to manage permissions, settings, personal data, linked persons, or other user-controlled actions. | Cannot change/delete/cancel/save/manage user-controlled information/settings. | Authentication requirement by itself. | 1. أرغب في الغاء موعد الجرعه الثانيه ولايوجد 2. لا يمكن حذف او تعديل المضافين قديما الغير مفعلين | 1. I want to cancel the second-dose appointment, but the option is not available. 2. Previously added inactive persons cannot be deleted or edited. |

**Note.** Operational definitions and inclusion/exclusion guidance are reproduced from the finalized codebook used in the independent 300-review validation. Illustrative excerpts are reproduced verbatim from the Sehhaty review dataset; No personally identifying information is included.
